# “Treat Me with Respect”: Older Sexual Minority Men’s Interactions with Healthcare Providers

**DOI:** 10.1093/geroni/igaf122.683

**Published:** 2025-12-31

**Authors:** Mekiayla (Meki) Singleton, Jennafer Kwait, Deborah Konkle-Parker, Deanna Ware, Michael Plankey, Janet Turan, Katherine Wu, Mackey Friedman

**Affiliations:** Rutgers University, Newark, New Jersey, United States; Health Research & Policy Institute at Whitman Walker Institute, Washington, District of Columbia, United States; University of Mississippi Medical Center, Jackson, Mississippi, United States; Georgetown University Medical Center, Washington, District of Columbia, United States; Georgetown University, Washington, District of Columbia, United States; The University of Alabama at Birmingham, Birmingham, Alabama, United States; Johns Hopkins University, Baltimore, Maryland, United States; Rutgers University, Newark, New Jersey, United States

## Abstract

Engagement with the healthcare system, including interactions with healthcare providers, can have a critical impact on health outcomes. For marginalized communities, experiences with healthcare providers may be more challenging to navigate. The purpose of this study was to explore older sexual minority (SM) men’s experiences of their interactions with healthcare providers. Data for this study originated from the MACS/WIHS Combined Cohort Study (Stigma and Non-Communicable Syndemic Sub-Study). Participants (N = 887) were asked to use open-text responses included in a survey to describe times when they felt a) the most comfortable and b) least comfortable in their interactions with a healthcare provider. Data consisted of unstructured, free-text responses, which was analyzed using content analysis and organized into themes. The average age of participants was 60.7 (SD = 12.7) years; 69% identified as White, 10% were Hispanic; and 54% were living with HIV. Results showed that themes describing most comfortable interactions included: 1) having a provider that “listens and is attentive”; 2) “takes questions and concerns seriously”; 3) “discusses personal issues outside of health”; 4) “cares for the person as a whole”. Themes describing least comfortable interactions included: 1) “experiencing bias” around sexual orientation and HIV status and 2) “negative provider demeanor/attitude”. Outcomes from this work highlight that for older SM men there are specific characteristics that support feeling comfortable when interacting with a healthcare provider. These findings provide useful information that can inform and guide the implementation of policies and procedures in healthcare settings that serve a diverse older adult population.

